# Immune Response of Amebiasis and Immune Evasion by *Entamoeba histolytica*

**DOI:** 10.3389/fimmu.2016.00175

**Published:** 2016-05-12

**Authors:** Kumiko Nakada-Tsukui, Tomoyoshi Nozaki

**Affiliations:** ^1^Department of Parasitology, National Institute of Infectious Diseases, Tokyo, Japan; ^2^Graduate School of Life and Environmental Sciences, University of Tsukuba, Tsukuba, Japan

**Keywords:** *Entamoeba histolytica*, cysteine protease, glycosidase, mucin, phagocytosis, oxidative stress, metabolism

## Abstract

*Entamoeba histolytica* is a protozoan parasite and the causative agent of amebiasis. It is estimated approximately 1% of humans are infected with *E. histolytica*, resulting in an estimate of 100,000 deaths annually. Clinical manifestations of amebic infection range widely from asymptomatic to severe symptoms, including dysentery and extra-intestinal abscesses. Like other infectious diseases, it is assumed that only ~20% of infected individuals develop symptoms, and genetic factors of both the parasite and humans as well as the environmental factors, e.g., microbiota, determine outcome of infection. There are multiple essential steps in amebic infection: degradation of and invasion into the mucosal layer, adherence to the intestinal epithelium, invasion into the tissues, and dissemination to other organs. While the mechanisms of invasion and destruction of the host tissues by the amebae during infection have been elucidated at the molecular levels, it remains largely uncharacterized how the parasite survive in the host by evading and attacking host immune system. Recently, the strategies for immune evasion by the parasite have been unraveled, including immunomodulation to suppress IFN-γ production, elimination of immune cells and soluble immune mediators, and metabolic alterations against reactive oxygen and nitrogen species to fend off the attack from immune system. In this review, we summarized the latest knowledge on immune reaction and immune evasion during amebiasis.

## Introduction

*Entamoeba histolytica* is an enteric protozoan parasite that infects humans, and is the etiological agent of amebiasis. Amebiasis remains a worldwide health problem accounting for up to 100,000 deaths annually (1, 2). Transmission occurs via ingestion of food and water contaminated with amebic cysts (1, 3, 4). In endemic areas, exposure can be extremely high: an annual incidence of 40% was estimated among children in an urban slum in Bangladesh (5). In some parts of Asia and Australia, amebiasis is endemic among men who have sex with men (MSM) and can be transmitted sexually (6–9). Majority of infections with *E. histolytica* remain asymptomatic, while ~20% of the cases develop clinical manifestations, such as dysentery, which is characterized by colonic mucosal invasion and tissue destruction (10). Invasive disease includes dysentery and extra-intestinal amebiasis, most commonly amebic liver abscesses (ALAs), which occur in approximately 1% of symptomatic cases in developing countries and around 17% in Japan (11, 12).

**Figure 1:**
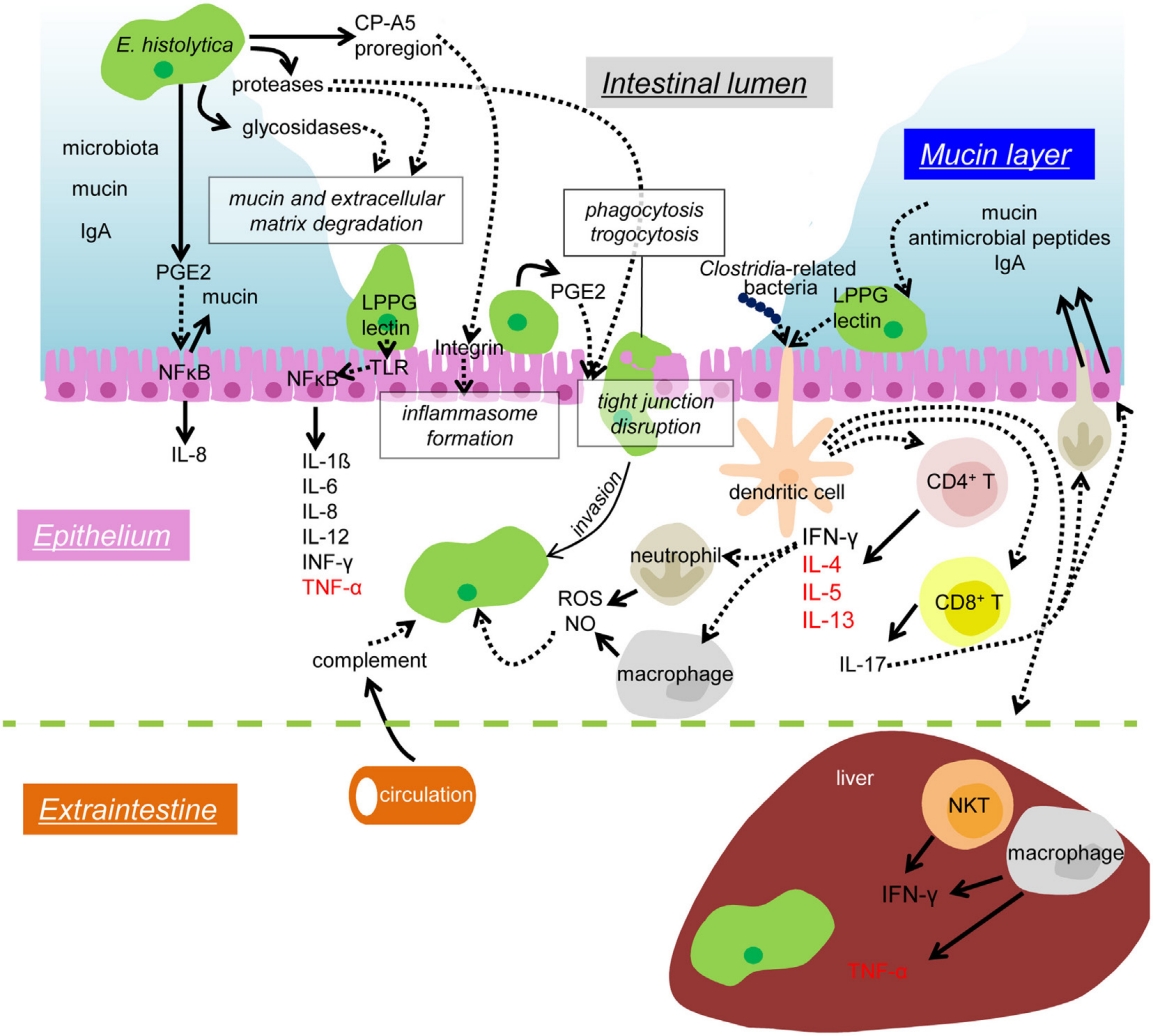
**Mechanisms of colonization and invasion by *E. histolytica* trophozoites and host immune responses to suppress and control amebic infection**. In the lumen of the large intestine, the IEC layer is covered by the mucus layer (blue), which contains secreted mucin and IgA from the host and commensal microbiota. Proteases and glycosidases secreted from the amebae are involved in the degradation of mucin and extracellular matrix. The pro-domain of EhCP-A5 binds to and activate integrin and enhances the inflammasome formation of leading to pro-inflammatory responses. PGE2 also secreted from the amebae causes mucin hypersecretion and depletion of mucin from the IECs. PGE2 also elicits signaling in a cascade leading to NFκB activation in the IECs and induces IL-8 secretion. The Gal/GalNAc lectin (lectin) and LPPG on the ameba’s surface binds to TLR2 and leads to NFκB activation and pro-inflammatory cytokine release for IEC. PGE2 also helps to disrupt tight junction function of the epithelium and enhances the amebic infiltration. Phagocytosis and trogocytosis are also involved in removal of host cells and invasion into the host tissue. Infiltrating trophozoites are attacked by complement from the circulation, ROS and NO from neutrophils and macrophages. The Gal/GalNAc lectin and LPPG activate CD4, CD8 T cells, and NKT cells, and, thus, enhances protective cellular immunity. CD4 T cells produce IFN-γ, IL-4, IL-5, and IL-13, and CD8 T cells produce IL-17. IL-17 induces neutrophil infiltration and enhances secretion of mucin, antimicrobial peptides, and IgA into the colonic lumen. When disseminated to the liver, the amebae are attached and removed by the dense mediated by IFN-γ secreted by NKT cells. TNF-α secreted from hepatic macrophages leads to abscess formation. Solid arrows depict secretion of soluble proteins and dotted arrows indicate interaction or signal transduction. Cytokines mainly beneficial for an elimination of the amebae are shown in black, while those involved in disease manifestations are shown in red.

**Figure 2:**
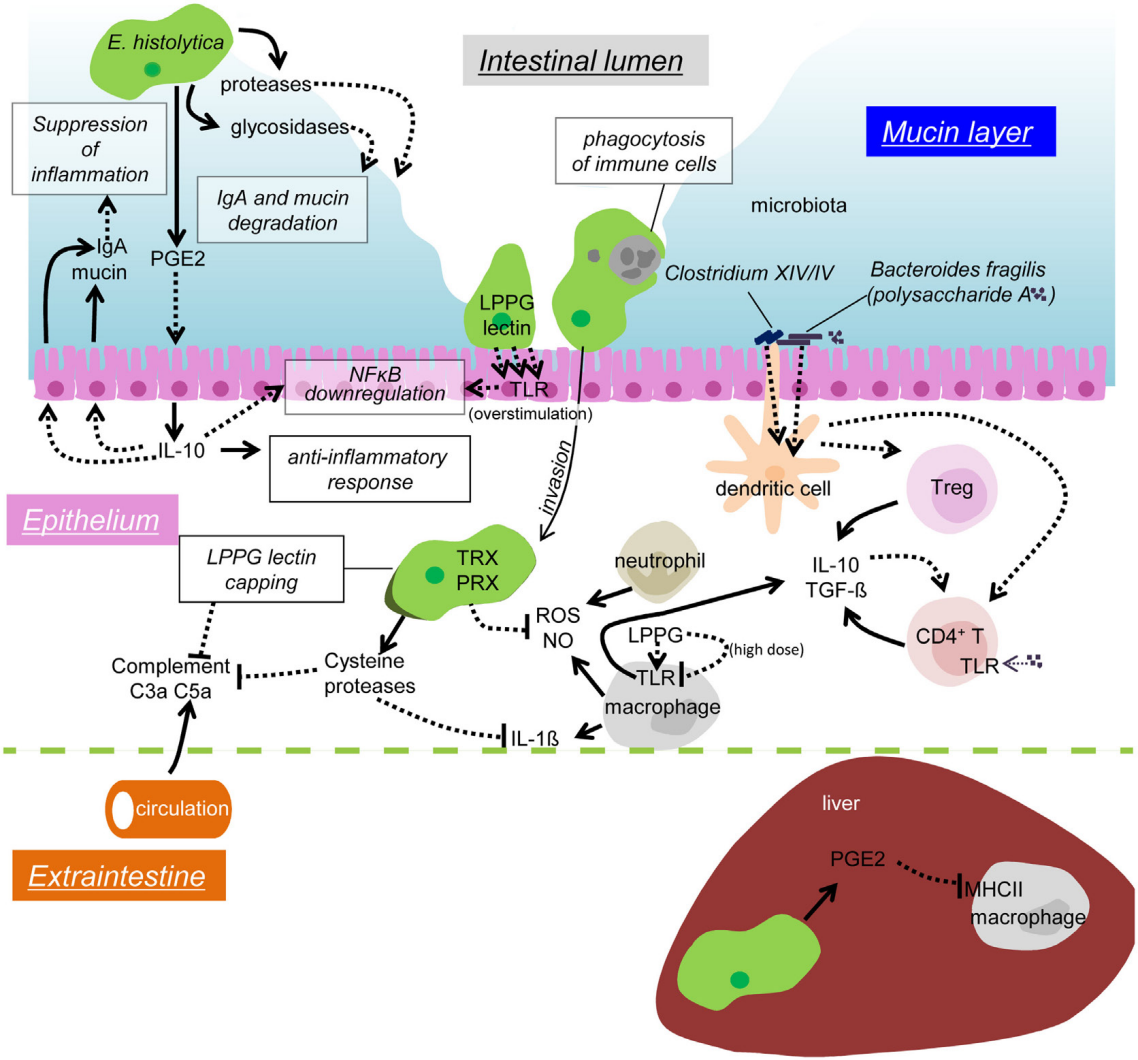
**Possible mechanisms of immune evasion during amebiasis**. Secreted or surface proteases of the amebae degrade IgA in the mucosal layer. PGE2 from the amebae induces IL-10 secretion from the IECs, and in turn stimulates mucin and IgA secretion, which likely prevents unnecessary inflammation. Overstimulation of TLR causes downregulation of NFκB activation. Removal of infiltrating immune cells by phagocytosis/trogocytosis helps to reduce immune responses. Some commensal microbiota, namely *Clostridium* XIV and IV groups and *Bacteroides fragilis*, induce Treg cells to downregulate immune responses. Polysaccharide A from *B. fragilis* binds to TLR2 on CD4 T cells and induces IL-10 production. The amebae in the tissues and the blood stream evade from complement by surface receptor capping (LPPG, lectin) and degradation of C3a and C5a by cysteine proteases. Cysteine proteases also degrade IL-1β, antioxidative stress defense by the TRX and PRX systems fends off the attack from ROS and NO from activated neutrophils and macrophages. LPPG binds to TLR2 on monocytes and macrophages, which leads to secretion of cytokines, including IL-10 and TGF-β. High doses of LPPG downregulate TLR2 gene expression in monocyte and cause negative feedback of protective immune responses. PGE2 from the amebae and the host causes downregulation of MHC class II expression on macrophages in the liver, which results in anti-inflammation.

When amebic trophozoites invade the colonic epithelium, they activate immune response in the human host. In order to survive in the host, the repression of host immune systems and the control of the environment of parasitism are crucial. For instance, during extraintestinal dissemination, the amebae must transiently survive in the blood vessels and the spleen, in which a network of immune cells and humoral factors are present, and the amebae are exposed to high concentrations of oxygen (*E. histolytica* are anaerobic or microaerophilic). To persist in such environment, amebae must subvert detection by antibody and complement, and resist oxidative and nitrosative attack. In this review, we summarize our current knowledge on immune response during amebic infection (Figure 1) and the parasite’s strategies to evade from host immune system (Figure 2).

## Immune Response During Amebic Infection

### Course of Amebic Infection

*Entamoeba histolytica* infection is initiated by parasite adherence to the colonic mucin layer. Trophozoites express a galactose and *N*-acetyl-d-galactosamine specific lectin (Gal/GalNAc lectin) on the cell surface and attach to host mucin and colonic epithelial cells (13). The colonized parasites are capable of extensive tissue destruction. Beside the pore-forming proteins, amoebapores (14, 15), hydrolytic enzymes, particularly cysteine proteases (CP), are considered to be essential weapons of the parasite to penetrate the epithelium and destroy components of the host’s extracellular matrix (ECM) (16–20). During and after penetration into the submucosal region, amebic trophozoites interact directly and indirectly with host immune and non-immune cells.

### Humoral Immunity

While the mucosal layer in the gastrointestinal tract generally serves as a primary physical barrier against intestinal pathogens, the intestinal immune response is the secondary defense to *E. histolytica* infection. Mucosal immunoglobulins (Ig) are the major component of the human intestinal defense mechanism (21). Among them, secretory IgA is one of the most abundant Ig produced by plasma cells and functions by preventing pathogens from adhering and removing the mucosal barrier (21). Haque and colleagues showed that the presence of Gal/GalNAc lectin-specific IgA antibodies in the stool correlated with reduced re-infection rates with *E. histolytica* in a study on susceptible children from Bangladesh (5, 22, 23). This implication was also confirmed with patients who had recovered from ALA. Increases in anti-Gal/GalNAc lectin IgA antibodies in post-ALA patients were associated with clearance of subsequent amebic infections, demonstrating that post-ALA patients developed a higher immune responsiveness and maintained immunological memory (24, 25). On the other hand, IgG levels have either protective or non-protective effects on the susceptibility to amebic infections depending upon major IgG subclasses induced by infection (i.e., IgG1 and IgG2 induced by Th2 and Th1, respectively) (26, 27).

### Cell-Mediated Immunity

Cell-mediated immune responses are also important for host defense against *E. histolytica*. During the initial stage of infection, intestinal epithelial cells (IECs) bind to and recognize the carbohydrate recognition domain of the Gal/GalNAc lectin via toll-like receptor (TLR)-2/4, which activates NFκB and leads to the production of inflammatory cytokines, including IL-1β, IL-6, IL-8, IL-12, IFN-γ, and TNF-α (28–30). IECs are the second line of barriers against pathogens after the mucosal layer and the first line of host cells to encounter microbial/parasite antigens, they express an array of pathogen recognition receptors (PRRs), including TLRs (31). IFN-γ is involved in clearance of infection, whereas IL-4 and TNF-α are associated with disease (32–35). In fact, IFN-γ production by peripheral mononuclear cells was shown to be correlated with protection from future *E. histolytica* infection in children (36) and the serum level of IL-4 was high in patients with invasive amebiasis (27, 37). It has been also shown that IFN-γ-producing CD4+ T cells and IL-17-producing CD8+ T cells are involved protection in vaccinated mice (38, 39). IL-17 plays multiple roles in protection against amebic infection, including induction of secretion of mucin and antimicrobial peptides, increase in IgA transport across the intestinal epithelium, and promotion of neutrophil infiltration (40–43).

IFN-γ-activated neutrophils and macrophages have amebicidal activity *in vitro* (44, 45). *In vivo*, neutrophils predominated in amebic lesions where the macrophages were infrequent, suggesting importance of neutrophils for clearance of amebae (46). Production of reactive oxygen species (ROS) and nitric oxide (NO) via NAD(P)H oxidase complex and iNOS, respectively, play critical roles in killing trophozoites (45, 47). In experimental ALA, protection was mediated by IFN-γ from natural killer T cells (NKTs), while TNF-α-producing macrophages increased tissue damage (32, 33). Taken together, both humoral and cell-mediated immune responses play important roles against amebic infection.

## Microbiota-Mediated Modifications of Parasite Virulence and Host Immune Response

### Microbiota Affects Energy Metabolism and Growth of the Amebae

The adult human intestine has trillions of bacteria composed of more than hundreds of species. Recent studies have suggested that the intestinal bacterial microbiota may influence the outcome of protozoan infections (48, 49). The growth and survival of *E. histolytica* trophozoites depends on nutrients from the host and the microbiota. The bacterial microbiota produces glycosidases that degrade complex polysaccharides into forms available for the absorption by the amebae and the host (50). Microbial glycosidase activity determines the levels of free colonic carbohydrates (the glycobiome). Thus, microbiota potentially influences central energy metabolism of *E. histolytica* trophozoites. Since *E. histolytica* has numerous glycosidases, including amylases, β-hexosaminidases, and lysozymes encoded in its genome (51–55), and can degrade a panel of polysaccharides to yield monocarbohydrates, the activity and regulation of amebic glycosidases also influence available carbohydrate concentrations.

### Microbiota Influences the Parasite’s Virulence

The commensal bacteria are generally protective against enteric pathogens. However, *E. histolytica* infection requires the presence of enteric bacteria. Germ-free animals were resistant to *E. histolytica* infection, but the introduction of a single bacterial species restored amebic pathogenesis (56, 57).

It has been shown that axenization (i.e., removal of associating bacteria) of xenically cultivated trophozoites decreases virulence, and incubation of axenic trophozoites with live bacteria restored virulence in a contact-dependent manner (58, 59). Incubation of *E. histolytica* trophozoites with the enteropathogenic *Escherichia coli* (EHEC) or *Shigella dysenteriae* increased amebic adherence to and cytotoxicity against MDCK cell monolayer (60). These observations indicate the enteric microbiota influence *E. histolytica* virulence during human infection (also see Perturbation of the Enteric Microbiota by *E. histolytica*).

The microbiota-dependent glycobiome has an emerging role in regulating the virulence of enteric pathogenic bacteria, such as EHEC, *Clostridium difficile*, and *Salmonella enterica* serovar Typhimurium (*S. typhimurium*) (61–63). EHEC has a fucose-responsive regulator of virulence genes, while *C. difficile* growth is promoted with high concentrations of free sialic acid reproduced by sialidase from associating bacteria (*Bacteroides thetaiotaomicron*). Similarly, the *in vivo* virulence of *S. typhimurium* was shown to be dependent on both fucose and sialic acid (63). It has been shown that glucose starvation enhances *E. histolytica* virulence, motility, and lectin expression via URE-3BP (64, 65). This finding suggests that the ameba has an ability to sense glucose (and possibly other monosugars) to modulate its virulence. The amebae and the bacterial microbiota influence each other by providing energy source and degrading available carbohydrates.

### Microbiota Affects Host Immune Response

Gut microbiota plays a number of physiological roles involving digestion, metabolism, extraction of nutrients, synthesis of vitamins, prevention against colonization by pathogens, and immunomodulation (66–68). It has been demonstrated that *Bacteroides fragilis* and cluster XIV and IV of *Clostridium* species induce the development of regulatory T (Treg) cells in the colon (69, 70). Treg cells have the ability to suppress inflammatory responses through the production of anti-inflammatory cytokines, including IL-10 and TGF-β, and are considered to be involved in self-tolerance (71, 72). The beneficial effect of *B. fragilis* depends on the expression of polysaccharide A, which is a unique surface polysaccharide that binds to TLR2 on CD4+ T cells (72). Microbiota-mediated immunomodulation is evident in several diseases, e. g., Rheumatoid arthritis, diabetes, obesity, and cancer (73–78). It seems that *E. histolytica* requires the intestinal microbiota for pathogenesis, and, conversely, the parasite also needs to disrupt the homeostasis of the microbiota during infection.

## Strategies for Immune Evasion of *E. histolytica*

### Disruption of Host Physical Barriers and Soluble Immune Mediators by Hydrolases

#### Glycosidases

Hydrolases secreted by *E. histolytica* trophozoites are involved in the elimination of immune cells and degradation and/or activation of soluble immune mediators, as well as disruption of the host gut and liver epithelia (29, 55, 79–84). The mucosal layer between the lumen and the epithelia forms a physical barrier. Degradation of carbohydrates in the barrier is crucial for the initiation of colonization by the amebae. Human intestinal mucus is mainly composed of highly glycosylated mucins (85). Among >20 human mucins, MUC2 is the major gel-forming mucin secreted by goblet cells of the small and large intestines (86, 87). When the amebae colonize the colonic epithelia, they binds to secreted mucin oligosaccharides with the Gal/GalNAc lectin and penetrate through the mucosal layer. In this process, the amebae decompose the mucin barrier to finally reach and subsequently attach on IEC (13).

Secreted proteins by *E. histolytica* trophozoites displayed glycosidase activities, including β-*N*-acetyl-d-glucosaminidase, α-d-glucosidase, β-d-galactosidase, β-l-fucosidase, and α-*N*-acetyl-d-galactosaminidase (88). Among these glycosidases, β-*N*-acetyl-d-glucosaminidase showed the highest activity (88, 89). Thus, β-*N*-acetyl-d-glucosaminidase activity likely have a central role in degrading carbohydrates on mucin and exposing its protein backbone (88). It was previously demonstrated that the amount of intracellular and secreted β-*N*-acetyl-d-glucosaminidase activity increased by complement in the serum (90, 91). Huldt et al. also suggested that hexosaminidase activity plays a role in the amebic virulence (90).

Recently, it has been shown that knock down of a β-amylase gene by siRNA caused reduction in the degradation of the mucosal layer and the invasion into the human colon in an *ex vivo* experiment (55). Furthermore, the β-amylase gene was found upregulated after contact with colon tissues (55). Mucin degradation by amebic glycosidases may also affect the central metabolism of the amebae *per se* and also the microbiota equilibrium in the colon since highly glycosylated mucin is a carbon source for the amebae and the colonic microbiota (92, 93).

#### Cysteine Proteases

The *E. histolytica* genome has ~50 genes encoding CPs (20), which likely reflects robust biological importance of CPs. Of these, however, only four proteins, EhCP-A1, EhCP-A2, EhCP-A5, and EhCP-A7, are highly expressed under culture conditions and altogether account for more than 90% of the proteolytic activity in trophozoite extracts (94). After mucin was digested by amebic glycosidases, the protein backbone of mucin is degraded by robust CPs. Altogether, these mucin-digesting glycosidases and proteases are the ameba’s first line strategy to overcome the innate defense of the mucus barrier.

As suggested by various studies, among the four major CPs, EhCP-A5 appears to play a pivotal role in virulence, including immunomodulation (80, 95–102). EhCP-A5 has a capacity to bind integrin via the RGD motif in the pro region, and elicits pro-inflammatory response in Caco-2 cells *in vitro* and the murine colon via NLRP3 inflammasome activation independent of the CP activity (100, 102, 103). CPs are also known to modulate cell-mediated immunity by activating pro-inflammatory cytokines and also modulate humoral immunity (see below).

#### Involvement of Glycosidases and Proteases for Extraintestinal Propagation

When *E. histolytica* trophozoites propagate extraintestinally, they take a route similar to that of cancer metastasis (104), which requires both glycosidases and proteases for the disintegration of the basement membrane and entry into circulation (105–107). In case of ALA, amebic glycosidases and proteases are also needed to survive in the blood vessels (see Degradation of Immunoglobulins and Complements), and to destroy Kupffer cells, the epithelial cells, ECM, and hepatocytes in the liver. Thibeaux and colleagues have recently demonstrated that EhCP-A5 secreted from the amebae activates host matrix metalloproteases (MMP), a well-known mediator of ECM degradation (84). Recombinant EhCP-A5 restored the invasiveness of the *EhCP-A5* gene-silenced trophozoites, suggesting that proteases from both the ameba and the host contribute to the tissue invasion process. In contrast to proteases, the roles of glycosidases in pathophysiology of amebiasis are not well demonstrated. It is evident in cancer metastasis that the level of serum β-hexosaminidase correlates with the likeliness of liver metastasis in variety of cancers, including colon, breast, stomach, pancreas, small bowel, kidney, testis, melanoma, lymphoma, and myeloma (108). Increased levels of tissue β-hexosaminidase were also reported for breast, kidney, pancreas, thyroid, colon, ovary, brain, salivary gland, stomach, and larynx cancers (109–112). Thus, it is conceivable by analogy that amebic glycosidases are involved in tissue invasion and extraintestinal dissemination.

### Degradation of Immunoglobulins and Complements

As described above, the major component responsible for the intestinal immune response against amebic infection is secreted Igs. It was demonstrated that anti-Gal/GalNAc lectin IgA reduces trophozoite colonization in the colon (5, 23, 25, 113–117). Intriguingly, *E. histolytica* surface-associated CP [most likely EhCP-A5, (118)] cleaves human IgA (16, 119). Amebic CPs are capable of cleaving both isotypes, i.e., IgA1 and IgA2 (119, 120). Furthermore, amebic CPs can also inactivate circulating IgG and, thus, believed to be involved in the survival during tissue invasion and extraintestinal propagation (18). Degradation of IgG in the blood could prevent activation of the classical pathway of the complement system and immune cells that harbor Fc receptors (19).

When the trophozoites are exposed to the intravascular immune system, complements are the major component that mediates trophozoite destruction. *E. histolytica* trophozoites evade from a complement attack by cleaving and inactivating anaphylatoxins C5a and C3a with CPs (79). C5a and C3a are potent activators of inflammation and enhance the release of histamine from mast cells, lysosomal enzymes from leukocytes, and pro-inflammatory cytokines, including IL-6 and TNF-α, from macrophages (121–123). C5a and C3a also increase vascular permeability and attract immune cells (122, 123). Reduction of these anaphylatoxins detracts from immune detection of the amebae in the blood and reduces inflammation in amebic lesions. It also partially explains the lack of severe inflammation in advanced colitis and ALA region.

### Degradation of Cytokines

Cysteine proteases are also known to modulate cell-mediated immunity by activating pro-inflammatory cytokine IL-1β and inactivation of pro- and mature IL-18 (82, 124). It is not concluded, however, if these changes are protective against or deleterious for amebic infection.

### Cell Surface Decorations to Evade Host Immunity

#### Glycosylphosphatidylinositol-Anchored Proteins

*Entamoeba histolytica* is also capable of evading from complement attach by decorating their surface with glycosylphosphatidylinositol (GPI)-anchored proteins. GPI is a glycolipid required for anchoring many proteins and glycoconjugates to the cell surface in most of eukaryotes (125–127). *E. histolytica* trophozoites expose on their cell surface a complex GPI-anchored glycoconjugate, designated lipopeptidophosphoglycan (LPPG) (128, 129). LPPG on the cell surface is a component of glycocalyx that is composed of oligosaccharides of glycoproteins and glycolipids and afford trophozoites protection by creating an impervious layer to complement (130, 131). It was demonstrated that complement-susceptible *Entamoeba dispar* trophozoites possess a much thinner structure of LPPG-containing glycocalyx, which is consistent with the premise that LPPG is important for the evasion from complement (130). It is also known that antibody against human CD59, a cell surface protein that prevents auto-lysis by inhibiting the formation of the membrane attack complex (MAC) antibody cross-reacts with Gal/GalNAc lectin and a 21 kDa surface protein (132, 133). Later, it was shown that the Gal/GalNAc lectin contains a CD59-like region on the cell surfaces that prevents MAC formation (132). These data suggest that the Gal/GalNAc lectin is a cross-reactive CD59 homolog of the ameba and have a similar function as CD59. In agreement with these results, global inhibition of GPI-anchor formation leaves *E. histolytica* trophozoites susceptible to complement-mediated lysis (131). However, functionality of 21 kDa protein as an inhibitor of MAC formation and its molecular identity has yet to be elucidated.

#### Surface Receptor Capping

Surface receptor capping is another strategy to hide from the immune system by disposing of the surface molecules that have been recognized by Igs or complements (134, 135). During cell movement, surface-bound immune complexes are translocated toward the uroid, where capped ligands accumulate (136). This polar re-distribution can be induced by concanavalin A (Con A) or anti-amebic polyclonal antibodies (137). It has been reported that serine protease, *E. histolytica* rhomboid protease (ROM1), is involved in the translocation of the complex to the base of the caps and subsequent release of the materials in the cap (135, 138). It is of note that ROM1 also cleaves the transmembrane domain of the heavy subunit of the Gal/GalNAc lectin (138). As the lectin heavy subunit is highly immunogenic, its release from the plasma membrane by ROM1 may interfere with host immune response directed to amebae.

### Killing and Phago/Togocytosis of Immune Cells

#### Contact-Dependent Cell Killing

Immobilization and killing of immune cells also serves as an ameba’s strategy for evasion from immune surveillance. Amebic trophozoites are able to kill a variety of cells, including neutrophils, T lymphocytes, macrophages, and a variety of tissue culture lines (116, 139–141). Adherence of the ameba triggers multiple intracellular events leading to cytotoxic effects to the mammalian cells. Such events include increased intracellular Ca^2+^, production of ROS, loss of membrane integrity, DNA fragmentation, phosphatidylserine exposure on the cell surface, and caspase-3 activation (116, 117, 139–144). It was reported that after host cell killing, *E. histolytica* preferentially ingest the dead cells (117, 140, 143). This observation is consistent with the theory that clearance of dead cells and debris by phagocytosis helps to minimize pro-inflammatory responses (145, 146). A phagocytosis-defective line of *E. histolytica* apparently showed decreased virulence *in vitro* and *in vivo*, suggesting a potential causal link between phagocytosis and virulence (147, 148).

Huston and colleagues demonstrated that *E. histolytica* preferentially ingests apoptotic Jurkat cells via recognition of phosphatidylserine and collectins (140, 149). Amebic calreticulin was found to be the surface receptor for host C1q, and required for phagocytosis of apoptotic cells, but it did not directly mediate cell killing (150). A few recent studies have started to unveil the detailed molecular mechanisms involved in the ameba phagocytosis (151, 152). However, the molecular events that take place in host immune cells in particular to suppress (or augment) immune response, together with a missing link between the surface receptor to the internalization machinery, remains totally unknown.

#### Trogocytosis

Ralston and colleagues have recently reported *E. histolytica* trophozoites ingested pieces of intact living cells via trogocytosis (*“trogo”* = nibbling) (153). When trophozoites were incubated with a combination of live and pre-killed host cells (Jurkat T cells), the live cells were ingested by trogocytosis, while the pre-killed host cells were ingested as a whole by canonical phagocytosis. Trogocytosis is an active process that resembles phagocytosis in some ways, i.e., it requires physiological temperature, actin rearrangements, Gal/GalNAc lectin, C2 domain-containing protein kinase, and phosphatidylinositol 3-phosphate kinase signaling, and it is accompanied with a rapid rise in intracellular Ca^2+^ concentrations. Trogocytosed host cells finally were killed. Trogocytosis of murine IEC was also evident in the *in vivo* animal model, suggesting that both trogocytosis of live host cells and phagocytosis of dead cells are important for pathogenesis and sustained parasitism of *E. histolytica*. Since amebic contact can potentially results in multiple outcomes: apoptosis and necrosis, followed by phagocytosis, or trogocytosis, it remains to be elucidated what factors and conditions differentiate these distinct manners of killing and ingestion of target host cells.

### IFN-γ

*Entamoeba histolytica* regulates IFN-γ for survival in the host. In CBA mice, which are susceptible to *E. histolytica* cecal infection, the amebic infection led to upregulation of Th2 (IL-4, IL-5, and IL-13) and Th17 (IL-17) cytokine responses, while Th1 cytokines, IL-12p35 and IFN-γ, were suppressed (154). This indicates that suppression of INF-γ causes susceptibility of amebiasis. From cohort studies in Bangladesh, susceptible children with malnutrition showed lower IFN-γ levels (36, 155). Analysis of asymptomatic carriers of *E. histolytica* showed that carriers had higher levels of IFN-γ, while patients with invasive amebiasis displayed higher levels of IL-4 (35). The significance of IFN-γ in susceptibility is also implicated for ALA. It is known that more than 80% of all ALA cases occur in adult males (156–158), and the male predominance is attributable to testosterone (159). Lotter and colleagues showed that testosterone inhibits IFN-γ secretion from invariant natural killer T (iNKT) cells stimulated by LPPG, a physiological ligand for CD1d (159). iNKT cells are a subset of NKT cells that recognize lipid antigens in the context of CD1d and produce IFN-γ and IL-4. *E. histolytica* LPPG is presented on CD1d to invariant TCR and activates iNKT cells in combination with TLR signaling. αGalCer, a CD1d agonist, stimulates production of both IFN-γ and IL-4, whereas LPPG induces IFN-γ but not IL-4 production (33). These data suggest that iNKT cells provide a link between innate and adaptive immunity due to their capacity to produce large amounts of IFN-γ and IL-4 that can bias the immune response into either a Th1 or Th2 direction. Production of IFN-γ helps clearance of *E. histolytica* infection and controls abscess formation, whereas an adequate level of IFN-γ reduces the trophozoite number and pro-inflammatory response at a low level, and may balance for trophozoites to survive.

### IL-10

It is known that anti-inflammatory cytokine, IL-10, plays a critical role to maintain the mucosal barrier. IL-10-deficient mice have compromised and highly permeable mucosal barriers and develop spontaneous intestinal inflammation in response to normal microflora (160). A murine amebic colitis model demonstrated that IL-10 from hematopoietic cells (CD4+ T cells) acting upon the non-hematopoietic compartment (IEC) is required for innate resistance to parasite invasion (161). Furthermore, it has been shown that IL-10 enhances MUC2 production, suppresses activation of antigen-presenting cells, induces B cell class-switching to IgA, has anti-apoptotic effects on IECs, reduces pro-inflammatory NFκB signaling in IECs, and promotes induction of CD4+ Treg cells (162–165). Interestingly, in asymptomatic carriers, no elevation of IL-10 level was observed. On the other hand, the IL-10 level was increased in dysenteric and ALA patients (27, 37). These studies indicate that invasion of the colon and liver by *E. histolytica* elicits an anti-inflammatory immune response and may successfully suppress immune reaction to the amebae. Altogether, the ameba needs to balance IL-10 and inflammatory cytokine levels to establish infection. It was shown that peritoneal monocytes and macrophages exposed to LPPG secreted TNF-α, IL-6, IL-8, IL-12, and IL-10 via TLR2 (166). It has been also shown that high doses of LPPG down-regulated TLR2 gene expression (166, 167). Thus, LPPG-driven signaling may activate a negative feedback loop that attenuates inflammatory responses. The mechanisms of the suppression of IL-10 production by the ameba remain to be elucidated (see below).

### Suppression of NFκB in IECs

*Entamoeba histolytica* trophozoites secrete materials that induce a protective response in human IECs (168, 169), the first line of host cells to encounter microbial antigens, via PRRs, including TLRs. Upon binding to their ligand, PRRs trigger activation of a transcription factor NFκB. Gut homeostasis requires continuous activation of NFκB by TLR signaling in response to intestinal bacteria (170), commensal microbes can also disrupt NFκB signaling to attenuate pro-inflammatory IEC responses (171). It has been shown that secreted components from *E. histolytica* trophozoites induce a protective response in human IECs that primed by macrophage secretions through suppression of NFκB via heat shock protein response and increase resistance of IECs to apoptosis (168). Thus, it appears that *E. histolytica* elicits a stress response to IECs and promotes a hyporesponsive state toward trophozoites. The amebic factors that induce NFκB suppression have not yet determined. The factors that activate TLR2, i.e., LPPG and Gal/GalNAc lectin, are candidates involved in this pathway (172).

### Prostaglandin PGE2

*Entamoeba histolytica* trophozoites produce and secrete prostaglandin 2 (PGE2), which have contact-independent effects on tight junction integrity and ion absorption. Secreted amebic PGE2 binds to prostaglandin E receptor 4 (EP4) on IECs, disrupts tight junctions, and increases luminal Cl^−^ secretion (173, 174). PGE2 secreted from the amebae elicits inflammatory response in IECs by increasing IL-8 production by IECs (173). PGE2 is a potent mucin secretagogue (175) that can overcome luminal barrier function by causing hypersecretion and, thus, depletion of the protective mucus barrier (176). On the contrary, it has been also reported that during invasive amebiasis, local PGE2 has anti-inflammatory effect. In animal model of chronic ALA, hepatic granuloma macrophages do not respond to IFN-γ and LPS and do not produce inflammatory cytokines, show decrease in MHC class II expression, and are unable to kill trophozoites (47, 177, 178). This suppression is local during chronic ALA and is directly caused by the parasite (47, 177). A culture supernatant and an unknown soluble protein component of *E. histolytica* trophozoites decrease class II major histocompatibility complex (MHC II) immune-associated (Ia) antigen expression through a PGE2-dependent manner (178). Inhibition of macrophage PGE2 synthesis can partially recover MHC II Ia expression and TNF-α expression (177, 178). However, inhibition of PGE2 synthesis does not recover iNOS expression or amebicidal activity in the deactivated macrophage (177). A continuous supply of parasite-derived PGE2 likely prevents iNOS expression and full recovery of MHC II and TNF-α, possibly through a concentration-dependent effect of PGE2. In short, ameba-secreted PGE2 represses inflammation in ALA, which is beneficial for survival, whereas it likely enhances destruction of the colon.

## Perturbation of the Enteric Microbiota by *E. histolytica*

It has been reported that *E. histolytica* infection alters the microbiota composition. *E. histolytica*-induced dysbiosis was characterized by fewer *Bacteroides*, *Clostridia*, *Lactobacillus*, *Campylobacter*, and *Eubacterium* species, and increased *Bifidobacterium* species (179). *In vitro* experiments have shown that *E. histolytica* preferentially ingest some bacterial species (59, 180). It is known that amoebapores, a family of the major pore-forming peptides, have differential activity against bacteria and eukaryotes (15). Furthermore, *E. histolytica* infection induces production of colonic antimicrobial peptides, while the trophozoites degrade them (181). A recent study has shown that dendritic cells from the mouse intestine where *Clostridia*-related bacteria colonized provide IL-17A-dependent protection against amebic colitis (182). Detailed molecular events remain to be elucidated, however, by examining how alternations of the microbiota modulate host immune responses against amebic intestinal infection. Altogether, microbiota can be modulated by amebic infection, and in turn concentrations of carbohydrates (and other compounds) that affect growth and virulence of the amebae can strongly influence outcome of infection. It remains to be elucidated whether and how the amebae modulate the intestinal microbiota for their survival and parasitism.

## Strategy for Oxidative Stress Management and Metabolic Control

### Lack of Respiration and Antioxidative Stress Management in *E. histolytica*

*Entamoeba histolytica* trophozoites are microaerophilic and consume oxygen. They tolerate low levels of oxygen tension. *E. histolytica* lacks a conventional respiratory electron transport chain that terminates in the reduction of O_2_ to H_2_O. However, it does respire and tolerates up to 5% oxygen in the gas phase (183–185). The parasite lacks most of the components of antioxidant defense mechanisms that are widely present in other prokaryotic and eukaryotic organisms, such as catalase, peroxidase, glutathione, and the glutathione-recycling enzymes glutathione peroxidase and glutathione reductase (184, 185). However, during tissue invasion, trophozoites must fend off reactive oxygen and nitrogen species produced by activated immune cells through the respiratory burst. Thus, trophozoites must use antioxidative stress defense to survive immune surveillance.

### Anti-Oxidative Stress Response Contributes to Immune Evasion in *E. histolytica*

*Entamoeba histolytica* trophozoites contain high levels of cysteine, instead of glutathione, as the major thiol in the cell. They possess several enzymes to defend from oxidative stress, such as peroxiredoxin (Prx), superoxide dismutase, flavoprotein A, ferredoxin, thioredoxin (Trx), and Trx reductase (186, 187). The Trx/Trx reductase system is crucial for buffering sensitive proteins under oxidative stress (188). The amebicidal drugs, metronidazole and auranofin, are known to disrupt Trx (189, 190). Interestingly, the oxidative stress increases *E. histolytica* virulence. It has been shown that oxidative stress causes upregulation of a stress-induced adhesion factor and a phospholipid transporting P-type ATPase/flippase (187). Both genes are involved in adhesion and phagocytosis. Oxidative stress also alters metabolic flux, including glycerol and chitin biosynthesis, potentially triggering encystation (191). Furthermore, it has been shown that *E. histolytica* (HM-1:IMSS) responds more strongly to oxidative stress than *E. dispar* and *E. histolytica* non-virulent Rahman strain, and surface localization of Prx in HM-1:IMSS is associated with virulence (186). Altogether, antioxidative defense mechanisms in *E. histolytica* are associated with pathogenesis. For more details on the antioxidative management in *E. histolytica*, a recent review should be consulted (192).

## Conclusion

Our understanding of molecular mechanisms of the parasite’s pathogenesis, such as adherence to host cells, induction of apoptosis, degradation of mucin and ECM, tissue invasion, and phago/trogocytosis of host cells, has greatly advanced in recent years. So have mechanisms of immune evasion, such as induction of IL-10 and suppression of INF-γ, degradation of Igs, complement, and pro-inflammatory cytokines. In addition, defense against ROS and NO and evasion from antibody and complement-dependent killing also plays important roles in survival in the host. Furthermore, mutual signaling among the three domains in the complex network of the parasite, the human, and the microbiota with polymorphic genetic backgrounds affect outcome of amebic infection. Further research is needed to elucidate the molecular basis of the complex interaction in the intestinal ecosystem.

## Author Contributions

KN-T and TN have made substantial, direct, and intellectual contribution to the work and approved it for publication.

## Conflict of Interest Statement

The authors declare that the research was conducted in the absence of any commercial or financial relationships that could be construed as a potential conflict of interest.
